# What is civic participation in artificial intelligence?

**DOI:** 10.1177/23998083241296200

**Published:** 2024-11-01

**Authors:** Renée Sieber, Ana Brandusescu, Suthee Sangiambut, Abigail Adu-Daako

**Affiliations:** 5620 McGill University, Canada; 5620 McGill University, Canada; 5620 McGill University, Canada; 1439 University of California Berkeley, USA

**Keywords:** Artificial intelligence, machine learning, citizen, engagement, inclusion, empowerment

## Abstract

There are increasing calls across disciplines and sectors that the public should participate in decisions about the use of artificial intelligence (AI). Public input in governmental decision-making is particularly crucial to promoting a well-functioning democracy and mitigating harms from AI. However, AI’s opacity, mutability, and resource requirements impede meaningful civic engagement particularly in urban environments. Many prior systematic reviews of civic participation and AI draw on the smart city literature. However, several other disciplines influence civic participation in AI so a siloed disciplinary focus offers only partial guidance for participation’s future role in AI. Our multi-disciplinary analysis blends works in smart cities, and in public policy, communication and, importantly, computer science to reveal distinct and highly variable pathways for civic participation. We use a sequence of manual and automated steps to conduct a structured literature analysis beginning with over 3,000 articles. We categorize authors’ work on participation in AI into five themes: participation as a natural byproduct of automating government, participation facilitated through the medium of AI, participation in AI as quantification, participation as a technocracy of trust, and participation as meaningful. With few exceptions, authors seemed not to challenge the status quo nor diminish the authority of the experts. Authors focused on the processual without the influence and AI aided in that process orientation. We conclude that the future of public participation in AI requires careful attention to become meaningful including recognition of neoliberal intent and power differentials.

## Introduction

There are broad calls across disciplines, sectors and from civil society that the public should be engaged in government decisions to use artificial intelligence (AI). AI is envisioned as a fundamental enhancement to participatory democracy by “offer[ing] new avenues to re-enchant democracy and overcome some of its most pressing challenges” (Duberry, 2022: 195). AI technologies could strengthen government-citizen relations and empower citizens, for instance, by providing automated translation tools to facilitate engagement at city council meetings (Fernández-Martínez et al., 2018). Engagement also could improve the design and user experience of new AI applications (Leslie et al., 2021). Conversely, civic participation could impede benefits by failing to match the speed of AI development and engendering onerous regulations (Wilson, 2022). Significant evidence shows that AI amplifies bias and harm, increases surveillance of the already disenfranchised, and exacerbates systemic racism (Benjamin, 2019; Eubanks, 2018). Strengthened and ongoing citizen engagement potentially afforded by AI is thus more desirable than relying on elite representatives chosen through sporadic elections (Savaget et al., 2019) or consumer choice.

Sanchez (2023) offers recent examples of AI in cities. In Tuscaloosa, Alabama computer vision detected urban blight and optimized resource allocation and remediation. An Ector County, Texas school district employed GeoAI to promote social equity and community planning. Natural language processing (NLP) allowed planners in Long Beach, California to analyze public sentiments, providing valuable insights into community perceptions. These planners are using AI to engage the public and improve quality of life.

There is a long history of technically-enabled participation, through approaches like Public Participation Geographic Information Systems (PPGIS) (Sieber, 2006). More broadly in analytics and apps, one sees a role for citizen participation, for example, in calls for human-centered technological approaches that would “create a louder and stronger citizen voice” (Wilson et al., 2017: 286). AI is particularly attractive for civic participation because AI systems can work at scale, leverage massive amounts of data contributed by the public, rapidly synthesize unstructured and structured content from various sensors, and thus match the speed and complexity of urban changes.

To some AI-related participation seems odd because local community actions appear irrelevant in the face of AI applications that cross borders and confound legal and political regimes. Initiatives to fund, develop, regulate, and assess AI often occur at a national level under the umbrella of innovation policies, but still exert influence on city-level implementation. Cities remain responsible and accountable to citizens and underrepresented populations. In all jurisdictional levels, government can be a major funder and procurer of AI (Brandusescu, 2021). Given this, we argue that citizens should have a say in the way AI-based services and resources are allocated and used.

One factor complicating the examination of participation in AI is the vagueness of both terms. AI encompasses a bewildering array of data, software, and hardware (e.g., sensing technologies like the Internet of Things). AI includes predictive analytics, basic machine learning (ML) like logistic regression, as well as deep learning and generative AI. Each system differs in requisite skills and resources, in opacity of computational decision-making, and in emergent properties of heuristic systems. Deep learning is labeled unknowable to the overwhelming percentage of the population so it can be rendered “beyond scrutiny” for any meaningful non-expert engagement.

Participation is likewise vague, having been defined in myriad ways and manifested through community service, collective action, political involvement, or social change (Adler and Goggin 2005). Participation can involve activities like “reading newspapers [and] social networks and interpersonal trust to associational involvement” meant to influence people beyond one’s friends and family (Ekman and Amna, 2012: 284). The problem with participation is both its mutability and its potential for manipulation; it is “an infinitely malleable concept, [which] can be used to evoke—and to signify—almost anything that involves people. As such, it can easily be reframed to meet almost any demand made of it” (Cornwall, 2008: 269). Authors disagree on the level of political participation or whether citizen engagement should even embrace politics (Ekman and Amna, 2012). We focus on the interaction between cities and citizens on societal issues, to emphasize participation that involves a degree of political power and influence.^1^

Our paper responds to multi-disciplinary calls within the AI ethics and other disciplines for a “cultivation of an effective lingua franca [that serves] as a framework for civil society participation” (Prabhakaran et al., 2022: 6). A multi-disciplinary approach allows us to investigate how the views of individuals outside municipal government, such as computer scientists and legal scholars, view civic participation in AI. The approach also highlights the extent to which participation is shaped by fields outside of computational urban planning and management. The first vital step is to assess how researchers and practitioners describe participation in AI. Unlike other technologies, AI systems can be the reason for participation (e.g., use of facial recognition technology by police) and the medium of participation (e.g., use of chatbots to recommend public services to citizens). For policymakers and developers, participation may necessitate moving beyond stakeholder engagement, individual manipulation (i.e., nudging), or public provision of feedback, to actively engaging civil society in shaping AI decisions and design. We begin by examining past reviews of civic participation afforded by computational technologies and AI. Next, we outline a methodology for a structured literature analysis, which integrates an AI method with more traditional review methods. Five themes emerge from our literature analysis. We conclude by exploring the present and future tense of civic participation in and with AI.

## Reviews of civic participation through AI and related technologies

Past literature reviews can help us understand current practices and unmet needs when AI is associated with civic participation. Studies suggest a rapidly shifting field focused on the rising use of AI in municipalities coupled with objectives of increasing some form of civic engagement (De Sousa et al., 2019; Savaget et al., 2019). Many reviews reference smart cities as AI provides one tool among many to increase efficiencies and deliver municipal services.

De Sousa et al. (2019) are exemplary of these reviews, emphasizing the utility of AI by government function and domain (e.g., public administration, healthcare) because of AI’s benefits to citizens, communities, and government. Only two articles in their corpus directly referenced civic participation (i.e., use of AI to sense community well-being and AI as a method to enhance citizen participation). Sharma et al. (2020) systematically reviewed AI and effective governance and argued that AI could simplify bureaucracy, which would enhance citizen-state interactions.

When citizens are mentioned, involvement often differs from traditional models of participation (e.g., Arnstein, 1969). Reis et al. (2019) combined two separate analyses, searching on “government digitalization” and using “government AI,” to examine how AI would shape government. The authors found that government efforts to digitalize operations often leveraged AI to involve citizens in co-production and information sharing. Kwecko and Botelho (2018) reviewed technological approaches in smart cities, such as data mining and crowd computing, envisioning smart cities as centers of collective intelligence. Simonofski et al. (2017), distilled three categories of participants in the smart city: democratic participants, co-producers of value, and users of IT systems. In these reviews, co-production features prominently, evoking ways that computational technology repairs failures of prior innovations in governance, such as new public management.

Several reviews focused on civic participation at the national level or were agnostic to level of government. Zuiderwijk et al. (2021) reviewed AI usage in national government and identified seven forms of public governance, including participative governance, which encompassed public consultation and involvement in AI development. They recommended government engage with stakeholders (e.g., citizens) to address the public’s data concerns, related to privacy and security. Jobin et al. (2019) distilled 84 guidelines for ethical AI to eleven principles, including transparency, trust, freedom, autonomy, beneficence, and dignity. Participation was linked to transparency; empowerment was categorized under freedom and autonomy. Interestingly, the authors found a predisposition towards protection against negative impacts (non-maleficence) instead of beneficence that, according to their distillation, would encompass participation. Savaget et al. (2019) offered the only systematic review to explicitly explore political participation and its link to empowerment via AI. The majority of papers in their corpus avoided mention of how civil society could utilize AI to take more active roles in political affairs. Because Savaget et al. (2019) found no papers in their corpus covering the impacts of AI on civic participation in political or policy processes, they introduced a case study to explore empowerment.

Existing studies to identify generic challenges faced in AI usage tended to emphasize the potential for job loss, balancing of privacy and data acquisition, as well as implications for politics and ethics (Wirtz et al., 2019). With one exception (Savaget et al., 2019), the reviews did not challenge AI’s beneficial impact on participation despite political power implications (e.g., AI furthering marginalization). Explicit reference to implications for civic participation in AI remains systematically unexamined. Furthermore, reviews tended towards a single discipline (urban planning in Kwecko and Botelho, 2018) or omitted entire fields (computer science in Zuiderwijk et al., 2021) to focus on a specific element of discourse like ethics or governance. We argue that such narrowness can neglect the influence of tool-builders (i.e., computer scientists, software engineers) who are members of the AI governance ecosystem, especially when municipalities are more likely to outsource their AI development than develop the system in-house (Wan and Sieber, 2023).

In addition to broadening disciplinary inclusion, we ground our work with foundational research on civic participation (Arnstein, 1969; Cornwall, 2008; International Association of Public Participation, 2018) to distinguish authorial intent when authors invoke terms like participation, engagement, involvement, inclusion, and modifiers like meaningful. We also extend previous studies addressing related questions by using a more comprehensive set of search terms (cf. Savaget et al., 2019; Simonofski et al., 2017).

## Data and methods

**Figure 1.**
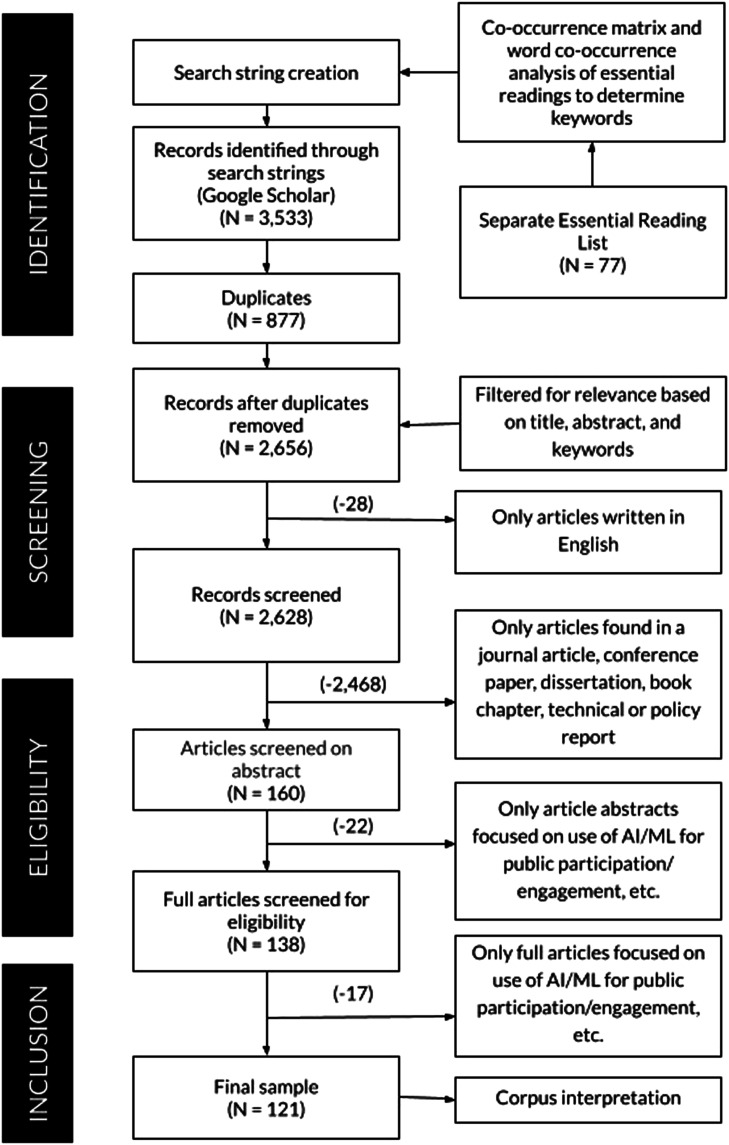
Overview of the methodological workflow.

We conducted a structured literature analysis using a series of search and refinement steps with manual and automated treatments. Our approach drew on Sacha et al. (2016), whose analysis began with an essential reading list, as well as papers such as Acciai and Capano (2021), who applied PRISMA (Page et al., 2021) to public policy. Our approach enabled us to ground and refine our search and minimize irrelevant results. The workflow is described in Figure 1.

Our goal was a multi-disciplinary analysis but we found a general search of AI and civic participation generated millions of documents. Therefore, in the Essential Reading List step, we seeded our search with well-cited works in AI ethics and participation. We converted and cleaned the full texts of the 77 articles. To inform our choice of keywords, we produced a co-occurrence matrix, which clusters word embeddings (Mikolov et al., 2013). That step guided the selection of search terms (e.g., was AI sufficiently comprehensive or did we need terms like chatbots?). The step resulted in a set of predominant technology terms, “Artificial Intelligence,” “Machine Learning,” “Algorithmic/ Algorithm(s),” “Automated Decision(s).” A set of participation terms also were extracted. These included “Democracy/Democratic,” “Governance/Government,” and variations on participation and engagement such as “Public/Political Participation/Engagement,” and “Citizen(s)/Civic Participation/Engagement.” In the next step, technology and participation terms were combined into search strings and used as queries for Google Scholar. This step matches other research, which achieved the best balance between sample coverage and relevance with both technology and public participation terms (De Sousa et al., 2019; Reis et al., 2019). The Corpus Collection step produced an initial search result of 3,533 articles. We removed 877 duplicates, which resulted in 2,656 articles.

**Figure 2.**
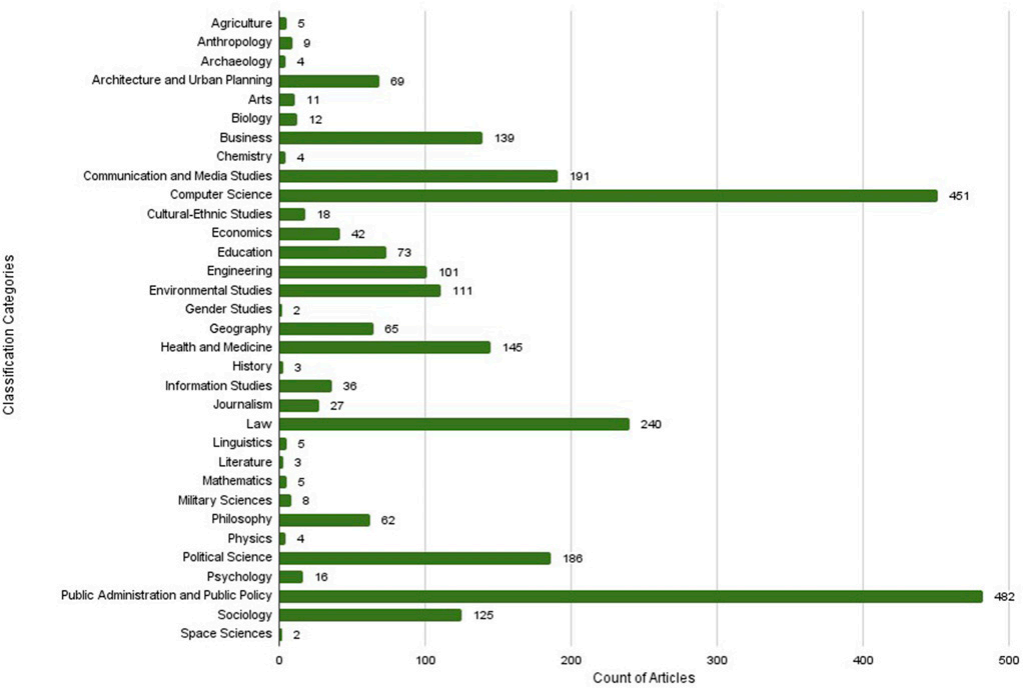
Corpus of 2,656 articles on AI/ML and participation from 1969 to 2022, classified using Classification of Instructional Programs (CIP) Canada 2000 categorization. Publicly available dataset at [https://osf.io/6tjb8].

We classified our corpus to demonstrate the numerous contributions across the discourse of AI and participation (Figure 2). The corpus spanned years 1969-2022, with the majority of articles published from 2010 to 2022. The Corpus Classification step revealed most articles from disciplines of public administration and policy, and law. Computer science was the second most prominent discipline, which suggests that developers didfinland not adhere to purely technical topics but also considered the participatory or governance aspects of their work. The disciplinary classification dataset is publicly available at [https://osf.io/6tjb8].

In the Corpus Filtering and Refinement step, we screened 2,656 articles based on titles, keywords, abstracts, and body content involving two sequential rounds of paper filtering and manual evaluation. Search terms presented their own challenges for inclusion/exclusion. Terms such as “technology,” “model” or “system” alone were too broad; whereas “smart technology” often implied AI or ML. We included articles that explicitly mentioned ML and participation (e.g., Sloane et al., 2020) but excluded ones that did not relate technology to public participation (e.g., “Public participation—its challenges in Hong Kong and some suggestions”), which were too broad (e.g., “Artificial intelligence and the future of humans”) or did not specifically mention AI, nor give the impression that AI would be the paper’s focus (e.g., “Public participation in urban design based on information technology”). Our final corpus of 121 articles was analyzed in the Corpus Interpretation step.

We convened as a group to identify themes around the subject of AI-related civic participation and classify articles into those themes. Most articles had mutually exclusive themes; however, some articles overlapped (see [https://osf.io/6tjb8]), which is expected in this kind of analysis.

Because civic participation is not value-neutral but evokes issues of power and influence, our theme identification was informed by critical discourse analysis (CDA) (Fairclough, 2001). CDA “is not a specific method of discourse studies but makes use of all relevant methods of the humanities and social sciences in the study of important social problems” (Van Dijk, 2015: 466). Table 1 shows the emergent themes result from the structured literature analysis. We draw inspiration from Mullet (2018)’s use of CDA to construct a similar table. A dataset, available at [https://osf.io/6tjb8], contains the thematic classification of each article. The first and second author classified and triangulated each article by theme(s). Subthemes were created to help authors operationalize the themes.

**Table 1.** Description of themes and how the articles were categorized with the subthemes. Includes example quotes illustrating aspects of the subthemes.^2^

Theme	Subtheme	Summary description	Examples
1. Participation as a natural byproduct of automating government	Motivations and drivers to adopt AI (efficiency, improvement of service delivery); often focus on neoliberalism (consumer, customer, co-production)	Adoption of AI in municipalities, other jurisdictional levels, where citizens tend to be indirect beneficiaries	“Most citizen-centric…initiatives are rooted in…a neoliberal conception of citizenship…that [are] not grounded in civil, social and political rights and common good” (Cardullo and Kitchin, 2019: 1)
			“The public sector seems to understand that the primary success of artificial intelligence is if the citizen is happy—not necessarily cost reduction” (Govloop, 2018: 5)
2. Participation facilitated through the medium of AI	Smart city applications; hardware/software (chatbots, sensors, Internet of Things, platforms); analysis (social media analysis, sentiment analysis, NLP, aggregations, synthesis); domains (e.g., land use, transportation). Often contains a methods section describing the application	Use of AI-enabled tools to augment or automate participation in a specific application(s)	“Enschede, a medium-size smart city in the Netherlands, has installed city traffic sensors that can register how often individuals visit the city...The city has developed smartphone traffic and cycling applications … that encourages individuals to cycle through alternative routes, rewards them for good behavior if they walk, cycle or take public transportation instead of driving” (Ranchordás, 2020: 21)
			“In the following, we will describe the NLP techniques that we have used in this project. As stated earlier, our objective is to apply these techniques to reduce the information overload that is inevitable in a large dataset of posts, and to implement them directly on the participation platform so that they can be used in real time to improve the effectiveness of the participation processes” (Arana-Catania et al., 2021: 9)
3. Participation in AI as quantification	Identification of types, number, counts, indicators or other quantification; reference to publics or humans in the loop or participatory processes; focus on assessment of utility or validity of AI-related tools used in participation; generalizing across applications	Assessment of the efficacy of AI-enabled tool or policy to broaden or augment participation. Assessment of quality of participation	“Our Algorithmic Impact Assessment Report helps affected communities and stakeholders assess the use of AI and algorithmic decision-making in public agencies and determine where—or if—their use is acceptable” (Reisman et al., 2018)
			“Another type of mini-public, citizens assemblies, are similar to citizens juries but bigger, involving more citizens (typically 50-250)….As with all other methods, they work best when resources are allocated to facilitate the involvement of a broad section of society” (Data Justice Lab, 2021: 13)
			“In contrast to resources such as fact sheets, guidelines, and checklists that aim towards standards and compliance, our AEKit embodies an agonistic politics aimed at direct political encounter” (Krafft et al., 2021: 9)
4. Participation as a technocracy of trust	Emphasis on role of government (e.g., in governance as experts, facilitators), AI frameworks that incorporate participation (e.g., Responsible AI, Trustworthy AI, AI ethics guidelines); specific references to trust, transparency, open data, literacy, explainability, privacy, simple forms of participation (e.g., feedback)	Focus on government’s direction of AI-enabled participation or AI policy and inclusion of the public’s voice	“AI is in some respects like the magical decision box. If we had such a device, we could employ it, and over time we would might to [sic] trust that its decisions were good” (Saetra, 2020: 9)
			“How can national strategic policies for AI enable and support trust between human and machine or AI (social trust), or citizen and government/nation-state (institutional trust)?” (Robinson, 2020: 3)
			Participants “expressed a preference for lower accuracy in some situations because they thought it would be easier to successfully appeal if the [AI] system were less trusted, resonating with the concept of ambiguity as a design resource” (Woodruff et al., 2020: 15)
5. Participation as meaningful	Emphasis on concepts of civic/citizen participation, empowerment, political power, citizen control, co-design, co-production, participatory design	Direct connection of AI-related participation to political power, empowerment and influence	“These questions also aid practitioners in assessing the extent to which a chosen participatory tactic or process can indeed meaningfully empower diverse stakeholders in their AI design” (Delgado et al., 2021: 1)
			“Overall, the individual belief-elicitation step of the framework—using both machine learning and explicit rule specification methods and visualizing the learned models—successfully enabled participants to build an individual model that represented their beliefs on how the algorithm should make a matching decision” (Lee et al., 2019: 18)

## Results from the structured literature analysis

Five themes emerge from the literature: (1) participation as a natural byproduct of automating government; (2) participation facilitated through the medium of AI; (3) participation in AI as quantification; (4) participation as a technocracy of trust; and (5) participation as meaningful.

### Participation as a natural byproduct of automating government

Despite our efforts to find articles that directly engaged with civic participation in AI, much of our corpus used participation as a justification for municipal adoption of AI. Citizens appear in these justifications as satisfied users as governments deliver services efficiently through automation (Battina, 2017). Citizen satisfaction with government presumably increases with greater responsiveness of services. Here participation is a transitive equation: AI adoption in government equals efficient service delivery, which equals improved lives of citizens, which equals citizen satisfaction with government, which equals participation. Indeed, governments may view the achievement of citizen happiness as a more important value for AI than efficiency, productivity, or cost reduction (GovLoop, 2018).

Numerous articles cast citizens as consumers, customers, or co-producers (e.g., Wilson 2022). Even the term “users” ties civic participation in AI to neoliberalism and reveals how values such as efficiency clashes with participation. Cardullo and Kitchin (2019: 1) argued that “most citizen-centric…initiatives are rooted in…a neoliberal conception of citizenship…that [are] not grounded in civil, social and political rights and common good.” Public engagement is reduced to a set of consumer demand signals with participation as a byproduct. Ironically, Wilson (2022) suggested that the costliness of adopting AI, for example, in terms of its potential negative impacts, might incentivize government to move beyond rhetoric to actually engage the public.

### Participation facilitated through the medium of AI

AI, particularly NLP, featured prominently in augmenting public engagement. Mehr et al. (2017) documented cases where government acquired AI to better serve citizens, using chatbots for citizen’s queries, AI-organized document retrieval, voice recognition to route citizen requests, and NLP-generated government responses. Tavanapour et al. (2019) sought to boost active participation in community planning by designing a chatbot to act as an “idea generator,” guiding discussions, posing questions, and bolstering participant comprehension. Arana-Catania et al. (2021) utilized NLP tools to analyze online comments in Madrid’s public consultation process, enhancing decision-makers’ efficiency in interpreting public sentiment. NLP, as demonstrated by Marmolejo-Ramos et al. (2022), served as a noise-reduction strategy, assisting facilitators in constructing meaningful narratives from extensive qualitative data during participation processes. NLP-driven engagement historically has faced criticism for simplistic and one-way communication, although newer iterations, like ChatGPT in urban planning, promise more nuanced conversations (Sanchez, 2023).

AI was reportedly used to classify user generated content on social media platforms to assess sentiment about the urban environment (Ghahramani et al., 2021; Milano et al., 2014). Here NLP shifts participation to a form of passive engagement, where AI infers the needs of the citizenry or reduces the requirement for citizens to appear in-person at town meetings (Arana-Catania et al., 2021; Gao et al., 2020). According to Lember et al. (2019: 1675), “The mere presence of citizens in public spaces provides the governments potentially valuable feedback.” Guenduez et al. (2020: 196-7) experimented with sensor-based data collection in the Swiss city of St Gallen: “By driving on a lit road, leaving a car in a parking space, using water, electricity or gas, or disposing waste, citizens communicate their needs to a certain extent through sensor systems.” They argued that “idle” or sensor-driven participation enabled a broader swath of individuals (e.g., foreigners, youth) to participate and improved the completeness of data collection.

AI’s role as a participatory medium reveals the impact of system design. Duberry (2022) explored NLP applications in opposing sides of a French political debate, highlighting the government’s platform (le Grand Débat National) and the “Gilet Jaunes” movement’s platform (le Vrai Débat). By featuring examples from differently configured instances of the same online participation platform, Duberry suggested that facilitators could use user interface and classification methods to mold the participation process. Consequently, the underlying architecture of AI systems, including data management, knowledge processing, and application layers (Androutsopoulou et al., 2019; Kontokosta, 2021) not only shapes the process but also influences the outcomes of civic participation.

### Participation in AI as quantification

Papers in the last theme emphasized the tool for a particular application; this theme interrogates generic tool development and refinement through types of quantification. A significant number of articles in the corpus relied on quantifying participation by identifying and counting relevant publics or designing assessment mechanisms. Various methods, such as citizen juries, assemblies, oversight mechanisms, participatory budgeting, and mini-publics, were suggested for public engagement in AI development and deployment (Ada Lovelace Institute, 2021; Balaram et al., 2018; Data Justice Lab., 2021). Participation as quantified measurement is not new so it is hardly surprising to see it emerge in the corpus. This theme of participation as quantification has faced criticism because a laser-focus on metrics has been considered a form of nominal or tokenistic engagement (Arnstein, 1969; Cornwall, 2008).

To address these critiques, new forms of measurement in participation in relation to AI have been developed, in which transparency is a necessary precondition and sometimes a sufficient substitute for participation. Methods like Algorithmic Impact Assessments (AIAs) are popular, often integrated into responsible AI initiatives in government (Harrison and Luna-Reyes, 2022). Watkins et al. (2021: 1011) considered AIAs an inclusive term for tools such as algorithmic audits, datasheets, “nutrition” labels, and model cards. AI Now Institute’s AIA Report focused on how “affected communities and stakeholders...assess the claims made about these systems, and to determine where—or if—their use is acceptable” (Reisman et al., 2018: 4). Assessments frequently measure whether public and impacted communities have provided feedback on potentially life-altering impacts (e.g., negative impacts of predictive policing). However, scoring public feedback may be a mere checkbox, as AIAs can overlook disparities in access and influence (Costanza-Chock et al., 2022).

Tool design of AIAs can limit civic participation efficacy, emphasizing easily quantifiable aspects and potentially discouraging more substantive involvement. Conflicts of interest in audit design, where auditors audit their funders, may narrow impact scopes (Costanza-Chock et al., 2022). Non-government AIAs, such as the Ada Lovelace Institute (2021)’s participatory data stewardship framework and the Algorithmic Equity Toolkit (AEKit) by Krafft et al. (2021) aim to broaden participation in AIAs and highlight additional impacts through their recommendations. The AEKit allows communities, and not AI developers, to define their own technical and social modes of failure.

Arguably, the goal of these designers is to improve outreach, broaden engagement to a greater cross-section of society, and reduce adverse impacts of AI. Despite challenges in quantifying nuanced responses, this theme underscores the importance of identifying who determines measurement criteria and outcomes in participation.

### Participation as a technocracy of trust

In numerous articles, participation in AI was frequently intertwined with trust in the government’s utilization of AI, encompassing considerations of citizens’ confidence in the ethical and responsible deployment of AI and the accuracy of its outputs (Prabhakaran et al., 2022). In more technocratic areas, trust is elided with social acceptance of AI. Illustrative of this theme is Robinson (2020: 3), who asked “How can national strategic policies for AI enable and support trust between human and machine or AI (social trust), or citizen and government/nation-state (institutional trust)?” Whether social or institutional trust, Robinson (2020: 7) argued that both require citizen participation as a precondition for trust.

Articles focused principally on government’s approach to AI without a substantial discussion of participation processes can all too easily become a technocracy or rule by experts, whether by human decision-makers or by algorithms (Lan et al., 2021; Saetra, 2020). In a technocracy, participation is shallow and unidirectional. Demands for participation by non-experts is seen by experts as a failure to adequately communicate policy to the public (Fischer, 1990), evoking a sentiment of “just trust us.” Harrison and Luna-Reyes (2022) were skeptical that public trust in AI would follow once citizens are adequately convinced or non-experts sufficiently educated, reinforcing the continued dominance of AI experts over the less informed. The ultimate technocracy in government would be an AI president, as posited by Wired Magazine in 2017 (cited in McEvoy, 2019), which exemplifies a technocratic ideal that views liberal democracy as flawed and perceives technology as the solution to societal challenges (König and Wenzelburger, 2021). Had we based our review only on social sciences (e.g., as opposed to humanities) articles, we likely would have seen a less didactic interpretation in which automation is trustworthy and participation is sometimes unnecessary or irrelevant.

What does civic participation regarding AI look like under a technocracy? It could be expert-led polls or feedback sessions (e.g., Tavanapour et al., 2019). Civic participation also is tied to learning campaigns or literacy projects, presuming that education could foster a better understanding of AI's benefits and alleviate public apprehension. Robinson (2020) emphasized education as a fundamental component of trust, citing initiatives like Finland's online course, Elements of AI. Without AI literacy, authors argued that AI could widen inequalities and exacerbate the digital divide (Kwecko and Botelho, 2018; Vanolo, 2016). However, concerns arose about the potential for AI literacy initiatives to exacerbate inequalities. Vanolo (2016) argued that education needed to extend beyond mere information dissemination; informing alone would not bridge a digital divide. Instead, citizens must be granted a meaningful political role to participate in AI decision-making. The goal of raising awareness or literacy may suit technocrats but render participation meaningless since participation becomes limited to informing, which Arnstein (1969) labeled a token form of participation. When examined through a CDA lens, even expert calls for fairness, accountability, transparency, explainability or trust could amount to what Sloane et al. (2020) call a kind of participation washing.

### Participation as meaningful

As suggested above, our working definition of civic participation in AI necessitates reviewing direct references to meaningful and co-occurring terms in the AI literature like fairness, justice, transparency, trust, explainability, and accountability. Of those who specifically mentioned meaningful participation, several authors drew on the most popular formulation of participation in urban planning: Arnstein’s ladder of citizen participation (e.g., Balaram et al., 2018; Delgado et al., 2021). Arnstein’s (1969) ladder utilized a hierarchy of participation *vis-à-vis* government, from forms of non-(manipulation) and token (informing) participation to more meaningful engagement (delegated power, citizen control). Cardullo and Kitchin (2019) extended Arnstein’s ladder to include roles, types of citizen involvement, and applicable political discourses (e.g., co-creation initiatives, human rights) within smart cities. They deemed sustained citizen control impossible with technologies like AI because communities mobilize on key social and environmental issues through the political process and policy creation, not through engaging in issue-agnostic technologies.

Few explicit definitions of meaningful participation “bring the lived experiences of people and communities who are affected by an algorithm to bear on the AIA process” (Ada Lovelace Institute, 2021: 25). These authors often referenced empowerment, defined variably as fostering a sense of control among participants or broadening participation to diverse groups (e.g., youth and seniors, non-experts and experts) (Nucera and Onuoha, 2018; People + AI Research, 2020). Some proposed a capabilities-focused participatory approach to empower marginalized communities throughout the design, development, and deployment of AI systems (Bondi et al., 2021; Nucera and Onuoha, 2018). Sloane et al. (2020) argued that any definition of participation in AI should be elevated to justice that is genuine and long term. That characterization requires mechanisms to investigate, contest, influence and even dismantle an AI system (Almada, 2019; Kalluri, 2020). Contra Cardullo and Kitchin (2019), authors, especially from the practitioner community, maintained that bottom-up engagement was crucial (Nucera and Onuoha, 2018). Therefore *meaningful* as a qualifier for participation in AI included different characterizations, from individual to collective, from providing a voice and building capacity, to a right of contestation and advocacy for social justice.

The concept of stakeholder engagement was prevalent. Stakeholder engagement, often interpreted broadly, tended to include all those with a stake in an AI system, from decision-makers, designers, and coders to government employees and community members (Figueras et al., 2021; Jobin et al., 2019). Loosely defining participation and participants could further mask power imbalances and lend the appearance of inclusion without meaningful representation.

Participatory design (PD) emerged as the predominant method for engaging the public in AI-related policy, emphasizing early individual engagement in the AI design process (Delgado et al., 2021). Costanza-Chock et al. (2022: 1580) found that any PD initiative required “investment in strategies to meaningfully engage community partners and support community-led processes for algorithmic accountability.” Lee et al. (2019)’s PD was among the most ambitious, engaging community members in defining optimization goals for algorithms and building computational models for decision-making. However, PD has faced criticism, with some arguing that participation often devolved into a form of labor or performative consultation, eliding crowdsourcing with meaningful participation (Sloane et al., 2020). Bratteteig and Verne (2018) conceded that AI could exceed the capacities of PD since even designers with sufficient knowledge and expertise could fail to anticipate emergent impacts and uses of AI, questioning the overall efficacy of methods for meaningful participation in AI.

## Discussion and conclusion

AI presents a complex frontier for civic participation, with significant disciplinary-bound concepts of participation. This diversity hampers a unified understanding of meaningful participation in urban settings or at any level of government. Confusion over participation terminology can create the illusion of public inclusion and democratic authority without granting participants the desired influence over political processes (Cornwall, 2008). We find that participation in the corpus lies in the eye of the beholder, defined and characterized to suit particular agendas, whether it naturalizes participation, is applied to specific domains, is abstracted as an assessment method, is associated with trust, or is linked to political power and influence.

Overall, we find a reluctance among the authors to explicitly include political power in civic participation related to AI (cf. Savaget et al., 2019) with polling and feedback often considered sufficient. Some authors believed that AI should naturally lead to more satisfied urban citizens or enhance participation. AI was largely viewed as a neutral tool to ease participation and was deemed relatively unproblematic, even as the wicked problems of urban dynamics added an extra layer of complexity (Goodspeed, 2015). Challenges in articulating and achieving meaningful civic participation persist and remain relevant in urban settings.

AI contains unique properties, such as opacity in outcome determination and heuristic algorithms constantly learning and recalibrating outcomes. Opacity can limit human ability to peer within the software and understand how outcomes are determined. This opacity can prevent human understanding of which input variables (e.g., age, gender, race, country of origin) dominate a classification output. Civic participation is complicated by AI if we assume that citizens’ identities and concerns are resistant to alterations of technology. However, algorithms can wield subtle influence. An algorithm can be used to select who has a stake in the purchase of an AI system; a chatbot can guide participation that nudges behavior towards acceptance; and an NLP system can synthesize public comments in a fashion favorable to a vendor (e.g., Ranchordás, 2020). This algorithmic exceptionalism exemplifies Gillespie’s (2014) “calculated publics,” positive feedback loops in which algorithms amplify and reflect the public back to the public, influencing how the public views itself, and hence, participation. Actors may not recognize being caught in this algorithmic capture nor identify effective escape routes.

Well-meaning municipalities may launch traditional participation initiatives for AI, such as stakeholder engagement and PD. Both may fall short of empowering participants due to divergent values and unequal resource distribution (Sambuli, 2021; Wilson, 2022). PD is widely endorsed, although very difficult to ensure meaningful participation. Because algorithms are heuristic, they can exist in a state of constant customization, a problem noted in Bratteteig and Verne (2018)’s critique of PD, when outcomes anticipated by participants do not match the outcomes of the deployed system. This suggests a classic Goldilocks issue of timing; participation should occur neither too early, nor too late. When considering a constantly evolving system, it is unclear how to identify the sweet spot for participation.

PD may require ascent from private developers, especially if cities have outsourced AI development because they lack the resources to develop AI systems or assess the impacts. In addition to its transjurisdictional nature, private sector firms are considered better able than the public sector to match AIs rapid innovation. The thought that public engagement should reside in the private sector differentiates AI (Groves, 2023; Sieber and Brandusescu, 2021) from older approaches like PPGIS (Sieber, 2006).

Authors spoke of the benefits of passive participation in AI as a way to alleviate the need for active participation. Passive participation in AI reflects a form of distantiated participation where government infers citizens’ intent or outsources civic participation. The citizen need not physically attend a city council meeting or communicate directly with elected officials. A disintermediation is likewise possible, where AI-enabled participation imagines a kind of Rousseauian public square. Here everyone can seemingly participate and then AI, deployed by the public or private sector, finds the signal amid the noise. ChatGPT and other text-to-text generative AI could further solve the need for participation “at scale” and synthesize public sentiment in a way that eclipses public opinion polling and passive harvesting. Customized outputs for the public delivered in a tone tailored to specific behaviors, can promote a kind of superficial participation, generating an AI hallucination of meaningful participation.

What does the future hold for AI-coupled civic participation? Calls for cities to automate decision-making can be over-determined. A city may invest in AI to demonstrate a competitive advantage and craft more public-private partnerships with AI firms; they may procure AI systems to save on labor costs. In a polarized world, AI promises to remove messy, subjective politics from policymaking (Wong, 2018). Generative AI furthers that promise with consensual syntheses. The over-determination driven by AI aligns with long-standing arguments in favor of technological progress (e.g., GovLoop, 2018) in which automation heralds greater efficiency, objectivity, rationality, and convenience (Arana-Catania et al., 2021). With this potential, AI better aligns with market-based principles of co-production, collective intelligence and other neoliberal visions of the public sector. This is why participation in AI requires careful attention to neoliberal intent and power differentials. Despite challenges that AI presents to civic participation and amid the hyperbole that AI may alternately destroy democracy or save it (Kaplan, 2020), the future demands a place for meaningful human-led participation.

## Data Availability

The four datasets that support the findings of this study are available at Open Science Framework and https://osf.io/6tjb8.

## Notes

1. Although considerable debate centers on the distinctions between engagement, involvement, and participation, as well as civic, citizen, and public participation, we use these terms colloquially and interchangeably.

2 Most articles had mutually exclusive themes: Theme 1 = 12 articles; Theme 2 = 22 articles; Theme 3 = 11 articles; Theme 4 = 29 articles; Theme 5 = 26 articles. As seen in [https://osf.io/6tjb8], 21 articles overlapped across two themes. With the exception of Theme 3, which showed a 45% overlap with other themes, we did not see a noticeable pattern in the overlaps.
